# Occupational Respirable Mine Dust and Diesel Particulate Matter Hazard Assessment in an Underground Gold Mine in Ghana

**DOI:** 10.5696/2156-9614-10.25.200305

**Published:** 2020-02-28

**Authors:** Martin K. Mensah, Kwadwo Mensah-Darkwa, Carsten Drebenstedt, Bright V. Annam, Edward K. Armah

**Affiliations:** 1 Department of Material Engineering, Kwame Nkrumah University of Science and Technology, Kumasi, Ghana; 2 Institute of Mining and Special Civil Engineering, Freiberg University of Mining and Technology, Freiberg, Germany; 3 Department of Applied Chemistry and Biochemistry, University for Development Studies, Ghana; 4 Department of Chemistry, Kwame Nkrumah University of Science and Technology, Kumasi, Ghana

**Keywords:** diesel particulate matter, respirable dust, silica, sampled exposure group, underground workers

## Abstract

**Background.:**

Underground miners can experience occupational health diseases due to exposure to particulate matter hazards.

**Objectives.:**

The aim of the present study was to examine occupational exposures of underground miners to dust and diesel particulate matter and to identify exposure groups with high potential to develop associated health effects due to the presence of dust and diesel particulate matter (DPM) hazards in an underground gold mine in Ghana.

**Methods.:**

Purposive sampling was employed using gravimetric air samplers over an 8-hour time weighted average period. The National Institute for Occupational Safety and Health (NIOSH) analytical Chapter Q and 5040 were used in determining crystalline silica dust and diesel particulate matter fractions, respectively. Structured questionnaires were administered to gather data on workers' level of awareness to dust and DPM exposures.

**Results.:**

It was found that 41% of the sampled groups were exposed to higher crystalline silica levels above the (NIOSH) permissible exposure limit (PEL) level of 0.05 mg/m^3^. For DPM, 49% of these groups had exposures above the Mine Safety and Health Administration (MSHA) PEL level of 160 μg/m^3^. Among the 94 mine workers who responded to this study, 62% were found to be aware of the presence and hazardous nature of silica dust, 28% had minimal knowledge and the remaining were found to be unaware.

**Conclusions.:**

There are varying levels of dust and DPM due to the presence of silica-bearing rocks, the production of diesel fumes and inefficiencies of available mitigation measures. Research carried out over the past decades has found confirmed cases of silicosis and lung cancer due to high dust exposure levels. Rock drillers, blast men and shotcrete operators were found to be exposed to higher levels of dust and diesel particulate matter and are at greater risk of silicosis.

**Participant Consent.:**

Obtained

**Ethics Approval.:**

This study was approved by the Ethics Committee of the Kwame Nkrumah University of Science and Technology, Ghana and carried out under full consent of the mining company under study.

**Competing Interests.:**

The authors declare no competing financial interests.

## Introduction

The mining industry serves as an economic driving force for countries with untapped natural resources.^1^ In Ghana, metals are the primary mined natural resource; the main extract is gold, followed by manganese and bauxite. The profitability of this sector has resulted in the growth of both legal and illegal mining companies.^2^ The former are well-controlled with regulations and safety measures in place for workers depending on their level of exposure to toxicants, whereas the latter remain anonymous owing to the illegality of their operations.^3^ The present study focuses on the legal gold mining sector in Ghana and the hazards it presents to human health. These hazards can be in the form of occupational injuries and/or ill health resulting directly from mining activities or their sequelae.^4^ The occurrence of mining hazards compares poorly to other well-established sectors such as manufacturing, rails and road construction.^5^ Air and noise pollution remain the most common hazards, owing to persistent excavating, drilling, bogging, crushing and hauling at the mining sites. The risk of developing disease from exposure to mining activities depends on the degree of inhaled dusts, duration of exposure, particulate size, ventilation and individual factors.^2^ In Ghana, there are ongoing processes to officially formalize an Occupational Health bill. This bill is expected to help develop additional health and safety standards that suit local scenarios. When put into place, it will help develop local exposure threshold limits, and enhance more stringent workplace protection in the management systems of mining industries. However, currently, internationally best practices in mining are adopted for use and supplemented with by-laws from the Inspectorate Division of the Minerals Commission for the regulation of formalized mines in Ghana. The studied gold mine was therefore under the supervision of these laws.

The main composition of respirable dust generated from quartz ore gold mines is crystalline silica, which has been implicated as the main toxicant in the pathogenesis of diseases within the mining field.^3^ Silica exists in two forms, crystalline and non-crystalline. The former is responsible for almost all of the harmful effects of silica owing to its ubiquitous nature, stability, aqueous insolubility and the presence of reactive oxygen species on its surface.^6^ Respirable dust has been defined as particles that penetrate into the gas exchange region of the lungs with particulate size less than 5 μ. Inhaled respirable crystalline silica is often the limiting factor in developing lung diseases associated with occupational inhalational dusts, such as pneumoconiosis or associated malignancies.^7^ The toxico-dynamics of inhaled silica are predominantly due to the reactive oxygen species mediating lipid peroxidation and genotoxicity among other aberrations.^8^ Exposure to respirable crystalline silica poses a significant problem, especially with regards to respiratory, renal, connective tissue, and malignant diseases.^9^,^10^ The International Agency for Research on Cancer (IARC) classifies silica as a potential human carcinogen.^11^,^12^ The consequences of exposure are prevalent in most ex-miners owing to chronic and acute toxicity effects after long latency periods. Respirable crystalline silica not only causes pneumoconiosis, but also predisposes individuals to infectious diseases such as tuberculosis.^10^

**Abbreviations**
*DPM*: Diesel particulate matter
*MSHA*: Mine Safety and Health Administration
*PEL*: Permissible exposure limit
*SD*: Standard deviation
*SEGs*: Sampled exposure groups

Diesel particulate matter Mine Safety and Health Administration Permissible exposure limit Standard deviation Sampled exposure groups Diesel particulate matter (DPM) is another composite toxicant released during mining activities which poses health risks to workers. Diesel particulate matter consists of carbon monoxide, carbon dioxide, hydrocarbons, oxides of nitrogen, ash, metallic abrasion particles, sulfates and silicates. In the early twentieth century, preclinical trials associated DPM with carcinogenic chemicals in mice, rats and hamsters, and human studies have shown similar results.^13^ Diesel particulate matter is considered by the IARCto be a group 1 human carcinogen.^11^ The unique nature of the diesel particulates, with a large surface area able to further absorb toxins, mutagenic and carcinogenic compounds such as polycyclic aromatic hydrocarbons, increases its likelihood of carcinogenesis. Diesel particulate matter has toxic effects on the lungs, heart, kidney, placenta, brain and liver.^9^ Respirable DPM is now an emerging concern in the mining workplace, and it is also of concern in domestic and industrial sites. Results from multiple studies at occupational sites have led to regulatory bodies considering the adoption of stringent threshold limit values or occupational exposure limits. Effective occupational health management programs for the mining industry focus on eradicating or limiting miners' exposure to DPM and to established safe levels within the PEL of 160 μg/m^3^ or less. This, according to the Mine Safety and Health Administration (MSHA) recommendations, can help prevent occupational diseases due to exposures to DPM hazards.^14^ The most common fuels used in mining are diesel and petrol, the latter has finer particulate matter and poses a lower risk in terms of deposition compared to the former. Since sulfur remains a characteristic component and pollutant in diesel fuels, the use of low-sulfur diesels and diesel oxidation catalysts are recommended, and although these do not produce a perfect safety margin, their toxicity is reduced. New technologies for diesel have dramatically reduced DPM by 99% in controlled studies in developed countries,^13^ but not in Ghana where traditional diesel exhaust is still employed. Although institutions have been set up to regulate exposure through monitoring and training, silicosis and DPM-associated diseases in miners still persist globally. This could be attributed to inadequate surveillance programs, as well as low occupational health and safety standards in some mines. Measures used to mitigate toxicity include the use of respirators, local exhaust ventilation systems, water suppression water blasts, as well as well-fitted and enclosed cabins. The occurrence of toxicities is prevalent in most developing countries like Ghana, where investors prioritize output over the safety of the workers. The present study was aimed at assessing the potential toxicity levels of respirable dust and diesel particulate matter among underground miners in a formalized gold mine in Ghana.

## Methods

An underground gold mining company in the western part of Ghana was selected for the present study owing to the accessibility and proximity to the mining site, large production scale and the company's interest and collaborative efforts in the research. Upon approval at the study site, and together with the occupational health team of the company, research protocols were refined between 2015–2016. The workers in the underground department were notified through toolbox meetings of the relevance of the study through the Health, Safety, and Environment department in advance. Miners with concerns about the study were given the opportunity to put them forward using the company's internal resolution procedure for redress where possible, however no objections were noted. The study was approved by the Ethics Committee of the Kwame Nkrumah University of Science and Technology, Ghana and carried out under full consent of the mining company under study.

### Sampling and data collection

Target groups were selected with reference to similar exposure groups and their job descriptions *(Table 1).* The sampling took into consideration the mining cycle and shifts system of workers since the target exposure groups were already known. Purposive sampling was used in selecting ninety-four (94) sampled exposure groups (SEGs) out of a total population of 144 for the study. Gravimetric air samplers (sampling train) were made up of a 10 mm nylon cyclone, pre-weighed polyvinyl chloride cassette, clipper and connecting tube. A charged constant flow pump (AirChek XR5000, model 210-5000, SKC Laboratories) calibrated to 1.7 L per minute was used. The 37 mm diameter low-ash polyvinyl chloride filter cassette was used for collecting dust samples, while quartz-fiber filters were used for collecting DPM samples.

**Table 1:** Sampled Exposure Groups and Job Descriptions

**Sampled exposure groups**	**Number sampled**	**Job description**
Truck operators	16	Haul waste and ore from underground
Shotcrete operators	4	Spray tunnels with cementing substances to help hold metal mesh and rocks mass together and prevent collapse
Diamond operators	8	General drilling
Solo operators	7	Drilling of production and service holes
Jumbo operators	14	Use mesh to support caved rock mass and drilling during development phase
Bogger operators	10	Clean development and production headings off ore and/or waste by loading them into trucks
Cubex operators	7	Drill service holes and production slots
Blastmen	8	Charge explosives in development phase, re-entry, carry explosives
Service men	14	Hang ventilation fans, extend water and air lines, and other utility maintenance activities underground
Supervisors	6	General monitoring and overseeing underground work activities

The target groups were identified and grouped with the help of mining supervisors and a survey of their work areas was conducted prior to the start of the present study. Not all workers were sampled due to shift and off-duty schedules. The sampling process was designed to take into account the mining system and work schedules available at the study areas. Participation was voluntary. Some workers were enthused about the activity since it had the potential to affirm previous complaints about particulate matter exposures. Each day the miners selected for the study were educated on the application of the sampling train in order to yield effective results. The pump was kept in a bag at the waist of each participant. The filter cassette and cyclone components were fixed at the breathing zone of the participants. Sampling time was 8 hours out of the 8–12 hours work shift. Post sampling calibration data were also determined on an 8-hour time weighted average for exposure rate computations. Gravimetric air samplers were used to collect dust and DPM samples from the breathing zones of the SEGs. Structured questionnaires, personal observations, as well as personal interviews were carried out to determine miners' awareness and knowledge of dust and DPM exposure effects. The questionnaire can be found in Supplemental Material. Workers who could not read/understand were assisted by the community and public relations team of the study company. Filling out of the questionnaire was done in workers' free time. The survey collected data on participants' work habits with regard to the use of recommended respirators as well as available mitigation measures in participants' work areas. Informed consent was obtained from all participants.

### Analysis and standard procedures

For compliances and authenticity purposes, the bagged dust and DPM samples were transported to an accredited laboratory (Gijima Holdings Proprietary limited) in South Africa for analysis. The National Institute for Occupational Safety and Health (NIOSH) analytical method 7500 was used in the analyses of the dust samples and the analytical method 5040 was used for the analysis of the DPM.^13^ The requisite quality control and quality assurance systems were employed in the analytical methods. Excel data analysis was used to run one-way analysis of variance to test for the level of significance among exposure groups. The MSHA permissible exposure limits (PEL) standards were used as the references for the study.

## Results

**Figure 1:**
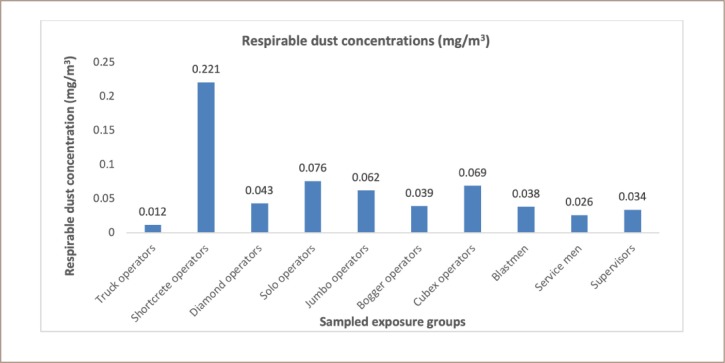
Potential average respirable dust concentrations across sampled exposure groups

Ten (10) different exposure groups were sampled for the analysis. Among the participants, shotcrete operators had the highest mean respirable dust exposure of 0.221 mg/m^3^. However, this exposure level was below NIOSH PEL of 5.0 mg/m^3^, though an incidental finding among the shotcrete workers established the presence of cement dust soiling the sampling train and pump bags. The next highest exposure level was solo drivers with a mean exposure of 0.076 mg/m^3^ and cubex operators at 0.069 mg/m^3^. The least recorded mean exposure value reported was for truck operators (0.012 mg/m^3^). Figure 1 shows the distribution of exposure among SEGs across all groups in the present study.

### Potential silica exposures

**Figure 2:**
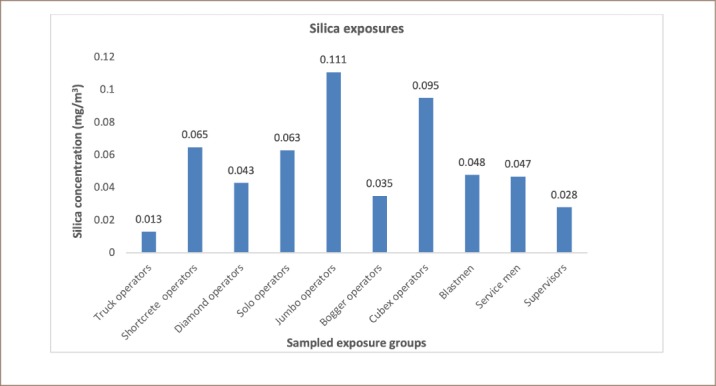
Potential average silica exposures across sampled exposure groups

In the present study, jumbo operators recorded the highest mean concentration of 0.11 ± 0.06 mg/m^3^ at about 122% over than the referenced PEL, followed by cubex operators (0.095 mg/m^3^). The highest exposures across groups were found in the order of jumbo operators, cubex operators, shotcrete operators and solo operators with recorded mean silica values of 0.111 ± 0.06, 0.095 ± 0.12, 0.065 ± 0.07 and 0.063 ± 0.06 mg/m^3^, respectively as shown in Figure 2.

To understand the exposure ranges based on safety margins and comparison to limits, three classes/bands (A, B, and C) were designated. Four out of the ten exposure groups were identified as class A (the most at-risk group and above the PEL), while the remaining were identified as classes B (moderate exposures below the PEL) and classes C (those that are generally not exposed to high silica concentration and values lie far below the PEL) as depicted in Table 2.

**Table 2:** Classes of sampled exposure groups in relationship to silica exposures

**Class A**	**Class B**	**Class C**
Jumbo operators	Blastmen	Supervisors
Cubex operators	Service men	Truck operators
Solo operators	Diamond operators	Bogger operators
Shotcrete operators		

### Diesel particulate matter exposures

**Figure 3:**
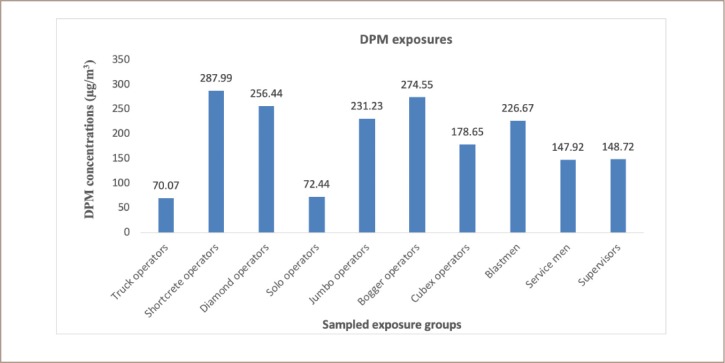
Potential average diesel particulate matter exposure across sampled exposure groups

The MSHA recommended PEL for DPM using its total carbon content over an 8-hour time weighted average is 160 μg/m^3^. Shotcrete operators reported the highest mean DPM value of 287.99 ± 142.80 μg/m^3^, followed by bogger operators with a mean value of 274.55 ± 127.18 μg/m^3^. Following was the DPM exposures which was reported by the diamond operators, jumbo operators, blast men and cubex operators at 256.44 ± 52.08 μg/m^3^, 231.23 ± 74.13 μg/m^3^, 226.67 ± 31.07 μg/m^3^ and 178.65 ± 135.80 μg/m^3^, respectively, as depicted in Figure 3. Analysis of variance showed a significant difference (p<0.05) in DPM exposure across SEGs. Results of the truck operators, solo operators, supervisors and service men were below the permissible limit with low exposure levels.

### Awareness of respirable dust, crystalline silica and particulate matter exposure by workers

Evidence of training and awareness on the investigated hazards were observed by means of toolbox meetings and safety timeout meetings. Worker knowledge of the presence, control and effects of DPM and dust hazards was also investigated. The scale for grading awareness levels in the present study is depicted in Table 3 which was adapted from our previous study.^17^ Out of the ninety-four (94) respondents, 62% were aware (good) of the presence and hazardous nature of the dusts while 28% had minimal (fair) knowledge of the detrimental effects. The remaining 9% were unaware (poor knowledge) of any adverse health effects that could result from dust exposures. In addition, 60% of the respondents were aware of the consequences of silicosis, a lung disease caused by silica dust exposure, while the remaining 40% reported vague knowledge. In the present study, 52%, 32% and 16% of respondents reported good, fair and poor levels of awareness to DPM at their working areas, respectively. Furthermore, 69% of the survey respondents mentioned that they generally felt uncomfortable using respirators and used them only irregularly. Factors such as difficulty in breathing during use, perceived low protection levels, and perceived absence of hazards were cited as reasons for either discomfort in or irregular use of assigned respirators.

**Table 3:** Grading Systems for Assessments of Target Group's Awareness of Dust and Diesel Particulate Matter Hazards (adapted)^17^

**Grade**	**Description**
Good	Ability to recognize a hazard and give/describe examples of sources as well as health and other implications of potential exposure to high levels. Stating/describing silicosis, lung cancer, tuberculosis among others as possible effects.
Fair	Ability to recognize the hazard and vaguely describe a related source and/or health implication to high levels apart from mentioning catarrh and/or coughing.
Poor	Loosely acknowledged the presence of the hazard, gave vague description, or did not name any potential source and/or health related effect to high exposure levels.

## Discussion

The aerodynamic size of aerosols determines their inhalability, i.e. the fraction of airborne mass that enters the nose or mouth during inhalation and the regional deposition of particles within the respiratory tract.^6^ The airborne concentration to which nearly all mine workers may be repeatedly exposed during a normal 8-hour work shift (or a 40-hour work week) is a time-weighted average limit and is expressed either in parts of vapor or gas per million parts of polluted air by volume at 25°C at 101.3 kPa pressure or in milligrams of pollutant per cubic meter of air (mg/m^3^).

Respirable dust particles are the main causes of pneumoconiosis, including silicosis. These dust particles cannot be easily seen by the naked eye; while observable dust mostly appears coarse in nature and comparatively harmless, the human eye is not a reliable means of detecting dangerous dust in the air.^10^ This poses a significant danger to underground mine workers. However, dust sampling instruments are capable of providing information on fine dust in air. Sampling instruments can determine whether respirable dust constitutes a danger to health, especially for dust particles of a size considered to be hazardous to human health, usually smaller than 5 to 7 μm.

As reported earlier in the present study, higher exposure levels of respirable dust among shotcrete operators could pose greater health risks to these workers, even though the mean values were below the PEL. This is because respirable dust includes crystalline silica components which can cause silicosis among mine workers carrying out underground operations. Minimal levels of respirable dust were reported in the case of truck operators, which could be due to lower or no direct exposures. Most truck operators carry out duties only when called in for service or may be involved in surface activities with less exposures to respirable dust. It is reported that there was a significant difference (p<0.05) between mean exposure levels among shotcrete operators (highest) and truck operators (lowest). A fair difference was reported for both solo operators (0.076 mg/m^3^) and cubex operators (0.069 mg/m^3^), indicating that their dust exposure levels are likely the same over time. The exposure levels below the PEL reported for supervisors, bogger operators, and truck operators could be attributed to the limited number of hours spent during underground operations compared to the other exposure groups. Most of their working hours were spent in a combination of field supervision and office work, resulting in relatively low hours in underground conditions and therefore low exposure risks. Previous studies have revealed the prevalence of silicosis due to exposures of 0.051 mg/m^3^ - 0.075 mg/m^3^ of silica^16^ similar to what is reported in this study for the range of 0.013–0.111 mg/m^3^.The high levels (0.063 mg/m^3^ - 0.111 mg/m^3^) reported in the present study suggest the risk of silicosis among mine workers if effective remedial measures are not implemented. In addition, a sensitivity analysis previously reported that exposure to crystalline silica is the most relevant parameter in cancer risk estimates for industry workers.^7^ A previous study reported that breaking down large deposits of silica-bearing rocks creates significant exposures to workers during underground mining operations.^17^ This is a major source of exposures for workers who may be unaware of the dangers this poses to their health. That study argued that stringent dust control measures were warranted to protect the health of underground mine workers. Out of the 94 workers sampled for silica exposures, 41% were exposed to higher levels of crystalline silica with reference to MSHA PEL value of 0.05 mg/m^3^. Over-exposure to crystalline silica dust has been classified as detrimental.^14^ A significant difference (p<0.05) was reported between the jumbo operators (highest mean value) and truck operators (lowest mean value). This indicates that exposure levels vary greatly across mining tasks and each job title has different exposure levels. The DPM is the total sum of all solid and liquid particles suspended in air, many of which have been reported as hazardous to workers.^13^ In the present study, six job titles were observed to have mean DMP values above the PEL of 160 μg/m^3^, including shotcrete operators, diamond operators, jumbo operators, bogger operators, cubex operators and blast men. A significant difference (p<0.05) was observed between the shotcrete operators (highest mean value) and the truck operators (lowest mean value) accounting for the maximum range of different exposure levels among the mine workers. A direct relationship between respirable dust and DPM was observed in the present study. The highest mean respirable dust exposure was observed among the shotcrete operators, which was also the case for DPMs for the same job title. The association in terms of the level of exposure encountered between the respirable dust and DPM values gives an indication that similar control measures could be adopted for both SEGs. Higher DPM is also linked to high diesel fumes generated by underground diesel equipment.^13^ With class A representing exposures exceeding the PEL, class B is a fragile zone (intermediate) and can easily oscillate between the two extreme classes of A and C owing to improved or deteriorated safety measures, respectively. Class C represents the least-at-risk group (far below the PEL) exposed to silica in underground working areas. While 100% of jumbo operators exceeded the referenced PEL of 0.05 mg/m^3^, 50% of shotcrete operators, diamond operators, solo operators as well as cubex operators were reported to be over-exposed, similar to results reported by Badri et al.^18^ There were significant differences (p<0.05) in exposure across the SEGs. In the present study, it was also observed that all miners used the same type of personal protective equipment for respiratory protection irrespective of the task and potential hazards that workers could be exposed to.

### Limitations

The sampled exposure groups were all men. Typically, miners in Ghana are men, which we note is a limitation of this study. We also recognize that the self-selection technique may bias the results, and is a limitation of the study.

## Conclusions

Silicosis and other silica-related diseases over the past decades have been attributed to respirable crystalline silica dust exposures in the gold mining industry, leading to mortality of workers globally. In addition, lower levels of exposure are associated with the increased prevalence of tuberculosis among miners. It was found in this study that the shotcrete operators recorded the maximum respirable dust and DPM levels of 0.221 mg/m^3^ and 287.99 μg/m^3^, respectively, among the SEGs, while the jumbo operators reported maximum silica content of 0.111 mg/m^3^. Higher DPMs were found to be due to diesel fumes generated during underground operations, and the results suggest that current protective measures for workers are unreliable and ineffective. The results of the present study suggest that these mine workers have higher risk of silica and DPM exposures at their working sites since most workers recorded values higher than permissible limits. It is recommended that available control measures be reassessed and improved in these mining sites, in addition to creation of a special treatment facility for affected workers.
