# Supplementary material for: Occupational Respirable Mine Dust and Diesel Particulate Matter Hazard Assessment in an Underground Gold Mine in Ghana
Source: J Health Pollut. 2020 Feb 28;10(25):200305. doi: 10.5696/2156-9614-10.25.200305 (PMC7058135; doi:10.5696/2156-9614-10.25.200305)
Supplement: Supplementary file 1 [file Mensah_Supplemental.docx]

**Supplemental Material**

**Questionnaire**

KWAME NKRUMAH UNIVERSITY OF SCIENCE AND TECHNOLOGY, KUMASI

This questionnaire is to enable Mr. Martin K. Mensah a student of KNUST to successfully conduct his research work on the topic “**Assessing the risk of exposure of underground mine workers to respirable dust and diesel particulate matter hazards, a case study of CGML**”.

**Please kindly fill / tick in the appropriate box/ space provided**.

1. Age……….**A**. 20 - 30 years **B**. 30 – 40 years **C**. 40 – 50 years **D**. Over 50 years
2. Job title……………………………………………
3. How long have you worked underground? **A**. 0 – 2 years **B**. 2 – 4 years **C**. 4- 6 years **D**. Over 6 years
4. When do you use your nose masks? **A**. When dust or DPM is seen **B**. When I enter the mine **C**. Never
5. How often do you use your nose mask (respirators) when at work?

**A**. As instructed **B**. As and when needed **C**. Never

1. Reason for answers in question **5** above? ............................................................................................................................

…………………………………………………………………………………………………………………………………………………….…………………………………..

1. Do you feel comfortable when using your nose mask? **A**. Yes **B** No
2. If no, Why? …………………………………………………………………………………………………………………………………………………………………...
3. Would you use your nose mask if supervisors were not enforcing use? **A**. Yes **B**. No **C**. Maybe.
4. Is dust present underground? **A**. Yes **B**. No **C**. No idea
5. Do you know or have you heard of any possible effects of respirable mine dust on your health? **A**. Yes **B**. No
6. If yes, please state (describe) them ………………………………………………………………………………………………………………………………………………………………………………………………………………
7. Is diesel fume present underground? **A**. Yes **B**. No C. No idea
8. Do you know of any possible effect of diesel fumes on your health? **A**. Yes **B**. No
9. If yes, please kindly state (describe) them …………………………………………………………………………………………………………..…….…………………………………………………………………………………………………
10. Have you ever been screened for any air-borne related diseases since you started working here?

**A**. Yes **B**. No

1. If yes, please specify ……………………………………………………………………………………………………………..……………..

What do you know about **silicosis**? ……………………………………………………………………………………………………………………………………………...............................................................................................
